# Strategies in Emergency Department-based COVID-19 Vaccination

**DOI:** 10.5811/westjem.2022.4.55043

**Published:** 2022-07-03

**Authors:** Anita Chary, Ynhi Thomas, Michelle Suh, Edgar Ordonez, Greg Buehler

**Affiliations:** * Baylor College of Medicine, Department of Emergency Medicine, Houston, Texas; † Baylor College of Medicine, Section of Health Services Research, Department of Medicine, Houston, Texas; ‡ Center for Innovations in Quality, Effectiveness and Safety, Michael E. DeBakey VA Medical Center, Houston, Texas

Emergency departments (ED) function as safety nets for vulnerable patient populations with limited access to healthcare. As such, EDs can play a critical role in advancing public health priorities such as vaccination for coronavirus 2019 (COVID-19) during the ongoing pandemic. Precedents for ED-based vaccination exist: established and routine ED-vaccination practices include tetanus prophylaxis in wound care^1^,^2^ and hepatitis B vaccination in cases of occupational exposures and sexual assault.^3^ Emergency departments have also previously delivered seasonal influenza vaccinations.^4^–^6^ Herein, we present our experiences providing COVID-19 vaccinations from the ED of an academic public hospital, based on a program developed immediately preceding the local surge of the COVID-19 Delta variant in a large metropolitan city.

The hospital where the ED COVID-19 vaccination initiative took place is a Level I trauma center with an annual ED visit volume of approximately 80,000 pre-pandemic; it is the central public healthcare facility for the city and surrounding county. Over half of the patient population is uninsured (53.6%) and over three-quarters are Black and/or Latinx (81.9%), with many lacking regular access to healthcare.^7^ It is notable that these racial and ethnic groups have been disproportionately affected by COVID-19 in the US.^8^ Structural determinants of inequity such as unjust policies, economic inequality, and discrimination are pervasive issues in our community that have adverse downstream effects on our patients’ social determinants of health.^9^

A recent study in this ED’s population showed a high prevalence of financial resource strain and lack of reliable transportation as barriers to healthcare access before the pandemic.^10^ As the Delta variant began to surge locally in July 2021, the county’s vaccination rate was 42%,^11^ far below the goal of the approximate 70% needed for herd immunity.^12^ This low vaccination rate reflects both the strength of the state anti-vaccination movement^13^ as well as local barriers to healthcare access.^9^ Minimal access to primary care, infrequent healthcare seeking, and relatively lower availability of vaccines geographically in racial/ethnic minority communities^14^,^15^ limit opportunities for indigent patients to receive COVID-19 vaccination. Mindful of this, our multidisciplinary team implemented an intervention with the goal of delivering equitable access to vaccinations.

From March 2021 to the present day, a leadership team of emergency physicians, ED nurses, and hospital pharmacists developed a protocol to offer the first dose of the two-dose Pfizer or Moderna vaccines or the single-dose Johnson & Johnson vaccine to ED patients likely to be discharged. This protocol was undertaken as a quality improvement initiative, rather than formal research overseen by the hospital system’s institutional review board. Eligible patients have not been vaccinated for COVID-19, do not have active COVID-19 symptoms, and have not had an allergic reaction to the mRNA COVID-19 vaccine or any of its components. The process is as follows:

1. The clinician evaluating an eligible patient offers the vaccination, discusses its benefits and riskswith the patient, and obtains informed consent for vaccination;
2. the evaluating clinician places an order in the electronic health record (EHR) for the vaccine type the patient elects;
3. the evaluating clinician communicates with the area charge nurse regarding vaccine administration.

Patients are monitored for allergic reactions for 15 minutes after receiving a vaccine dose. Through the EHR, a list of patients who elect to receive the first dose of the Pfizer or Moderna vaccine is generated, allowing a scheduler to contact them regarding follow-up appointments for second vaccination doses. If they prefer to self-schedule, patients also receive verbal and written information at the time of discharge about how to access our health system’s online patient portal, which allows them to choose any clinic or hospital location within our health system for a second-dose vaccination appointment.

The above protocol and brief materials to guide counseling about benefits and risks of vaccination were distributed to emergency physicians, advanced practice clinicians, and nurses by email in July 2021. The email included a screenshot of how to order the vaccine through the EHR and a PowerPoint slide of sample language about vaccine safety and efficacy. A reminder email followed approximately two weeks later. Members of the vaccination leadership team additionally performed ED administrative walkthroughs several times per week to encourage clinicians to offer vaccination and use the protocol above.

From July–December 2021, the ED administered COVID-19 vaccines to 357 patients. Of these, 39% received the Johnson & Johnson (n = 139), 46% received the Pfizer (n = 166), and 15% received the Moderna vaccine (n = 52). More than half of the vaccines were administered in July and August 2021, immediately prior to and at the start of the local COVID-19 Delta surge (n = 205, 57%). A total of 111 of ED staff clinicians participated in vaccine administration, with 84% of attending physicians (n = 43), 76% of advanced practice clinicians (n = 13), and 67% of resident physicians (n = 29) using the protocol.

As an ED administrative vaccine working group, we met biweekly to identify and discuss challenges associated with the ED COVID-19 vaccination initiative and potential strategies to overcome them. Clinicians’ time constraints represent a challenge to routinely offering COVID-19 vaccination to eligible patients. Determining a patient’s vaccination status and counseling about COVID-19 vaccination can take several minutes per encounter, as EHRs of COVID-19 vaccination across health systems are not always mutually accessible. Conversations about benefits and risks can also entail a time commitment, particularly as clinicians must address possible side effects of vaccines and often engage patients about publicly circulating misinformation and conspiracy theories.^16^ In light of these issues, emergency clinicians may deprioritize offering COVID-19 vaccination to eligible patients, particularly in the face of competing tasks when caring for multiple patients with conditions of varying acuity. Given the high volumes and crowding common in EDs,^17^ emergency clinicians value rapid disposition and may have concerns over potentially extending a patient’s length of stay by offering a COVID-19 vaccination. Vaccine administration and post-vaccination monitoring also presents additional tasks for emergency nurses and hospital pharmacists. Notably, vaccines are delivered from the hospital pharmacy, and delays in administration may occur when medications for critically ill patients are prioritized elsewhere in the ED. Similar operational challenges have been identified in ED vaccination campaigns for other infectious diseases.^5^,^6^

We have undertaken several strategies to overcome challenges related to clinicians’ time constraints. First, we encourage triage and medical screening staff to include a brief mention of a patient’s COVID-19 vaccination status in their documentation whenever possible, which can prompt subsequent evaluating clinicians to offer vaccination. Second, we encourage clinicians to identify and offer vaccination to ambulatory patients early on in their ED visit after addressing a patient’s acute medical concerns. When offered to patients waiting for laboratory or imaging studies, vaccination can occur in parallel with workup rather than adding to ED length of stay. Third, as ours is a teaching institution, patients may engage with a junior resident or advanced practice clinician fellow, senior resident, and attending physician during their visit. Flexibility in which clinician explores a patient’s receptivity to vaccination is essential, with the care team delegating conversation about vaccination to the clinician with greatest bandwidth at a particular time. We promoted these strategies at staff meetings in August 2021, but experienced severe staffing shortages in September 2021 that were associated with a decline in vaccine administration. Accordingly, we cannot offer that these strategies led to increased vaccination. However, our administrative team received endorsement of the second and third strategies from the 10 clinicians who vaccinated the most patients from July–December 2021.

Patient receptivity to COVID-19 vaccination in the ED varies, as in the United States more broadly.^18^ Publicly circulating misinformation about COVID-19 vaccines, conspiracy theories,^16^ and reports of rare side effects and prior pause in administration of the Johnson & Johnson vaccine discourage some patients from vaccination. Historical injustices that biomedical establishments have perpetrated against racial minorities are part of collective community memory and contribute to mistrust of medical personnel.^19^–^21^ Similar findings are documented about acceptability of vaccination in Black and Latinx populations elsewhere.^22^ For some of our patients, particularly among those who are ethnic minorities, clinical decision-making occurs through family discussions, and even if a patient desires vaccination, opposition from a spouse or adult child caregiver can lead the patient to decline.

There are no simple solutions to address these complex sociocultural and structural barriers, particularly in short encounters between a patient and an emergency clinician who lack a longitudinal therapeutic relationship. Through our working group meetings and discussions with emergency clinicians who vaccinated the highest number of patients, we identified several potential facilitators to vaccine acceptance. While we have not yet formally evaluated these strategies’ effect on performance or impact measures, we offer the following as areas of future inquiry.

First, when a clinician discloses their own experience of COVID-19 infection or the loss or suffering of loved ones, this can foster relatability and make an abstract concept concrete and immediate. Second, disclosing one’s personal or familial experience of vaccination as a clinician can encourage vaccination among patients—for example, establishing a shared identity as a parent living with unvaccinated children or wanting to protect an eligible teen attending school. Third, in our teaching institution, multiple clinicians can reinforce each other’s recommendations. Hearing the same messages about the benefits of vaccination outweighing risks from a resident physician, advanced practice clinician, and attending physician may help reassure patients about vaccine safety and efficacy. Finally, the notable diversity of our ED staff likely plays a role in fostering trust and opening conversations with our diverse patient population.

In the 2020 academic year, 60% of residents identified as underrepresented in medicine (URM), with 30% Black and 30% Hispanic. Of faculty, 34% identify as URM. While we have not formally studied these dynamics, some Spanish-speaking patients have commented to Latinx or Spanish-speaking clinicians that they are grateful for the opportunity to discuss the vaccine with their physician directly in their native language. Black patients have also expressed positive feelings about discussing vaccination with Black physicians. Given vaccine hesitancy in Black and Hispanic populations,^22^ it is notable that patients made these comments specifically about vaccination discussions, rather than other medical treatment and educational discussions for which they originally sought medical care. At the same time, literature does suggest that physician-patient racial concordance is associated with higher patient satisfaction regarding communication for other medical conditions.^23^ Physician-patient language and racial concordance is not feasible in all encounters, and clinicians may rely on interpreters and other means of establishing rapport. However, our experiences highlight the potential role of a diverse healthcare workforce to foster trust and improve perceptions of communication with racial/ethnic minority and limited English-proficiency patient populations.

It is worth noting that some of our ED patients quickly and readily accept vaccination when we offer it. These patients sometimes express that they had wanted to get the vaccine for months but had not done so due to limited engagement with healthcare establishments (eg, not having seen a clinician for years due to lack of health insurance). Some of our undocumented patients have disclosed fears that providing their personal or contact information could threaten their immigration status. In some cases, reassurance about contact information being used for follow-up vaccination led undocumented patients to feel comfortable to provide a phone number, either their own or that of a relative. In other cases, undocumented patients appreciated the option to self-schedule a second dose through the online patient portal.

We recognize multiple limitations in our approach. Our program was implemented during the Delta surge, which was quickly followed by the Omicron surge. During these times, our department and hospital system experienced severe staffing shortages, high patient volumes, and limited availability of information technology analysis services. As such, we were not able to track and are unable to provide information about the number and proportion of ED patients who were offered and declined COVID-19 vaccination. Schedulers for second-vaccine doses covered referrals from outpatient, urgent care, and emergency care in the hospital system, and information was not tracked about success rates of reaching ED patients needing a second vaccination or the proportion of ED patients who successfully received a second vaccination. We did not formally study the effects of our initiative on overcoming barriers to vaccination or of physician-patient racial or language concordance on vaccine communication and cannot provide performance or impact measures. Finally, because COVID-19 vaccination involves two doses and boosters, our experiences may not be applicable to vaccinations that either do not require follow-up dosing (eg, tetanus) or whose follow-up dosing is managed by occupational health entities (eg, hepatitis B).

Despite these limitations, we share our experiences in hopes that other EDs can benefit and capitalize on the unique safety net role that the ED plays in the US health system. COVID-19 vaccination from the ED can also pave the way for other important types of vaccination, whether broad initiatives such as seasonal influenza vaccination at the community level, or targeted efforts such as pneumococcal vaccination for individuals with sickle cell disease or for older adults. Emergency medicine is a specialty that prides itself on saving lives. Primary prevention through ED vaccination is a crucial way to accomplish this goal.

## References

[1] Centers for Disease Control and Prevention (2012). Updated Recommendations for Use of Tetanus Toxoid, Reduced Diphtheria Toxoid, and Acellular Pertussis (Tdap) Vaccine in Adults Aged 65 Years and Older — Advisory Committee on Immunization Practices (ACIP), 2012. MMWR Morb Mortal Wkly Rep.

[2] Miller T, Olivieri P, Singer E (2017). The utility of Tdap in the emergency department. The Am J Emerg Med.

[3] Pacella C (2021). Occupational exposures, infection control, and standard precautions.

[4] Buenger LE, Webber EC (2020). Clinical decision support in the electronic medical record to increase rates of influenza vaccination in a pediatric emergency department. Pediatr Emerg Care.

[5] Casalino E, Ghazali A, Bouzid D (2018). Emergency department influenza vaccination campaign allows increasing influenza vaccination coverage without disrupting time interval quality indicators. Intern Emerg Med.

[6] Ozog N, Steenbeek A, Curran J (2020). Attitudes toward influenza vaccination administration in the emergency department among patients: a cross-sectional survey. J Emerg Nurs.

[7] Harris Health System (2021). Harris Health Facts and Figures.

[8] Mackey K, Ayers CK, Kondo KK (2021). Racial and ethnic disparities in COVID-19-related infections, hospitalizations, and deaths: a systematic review. Ann Intern Med.

[9] Houston Health Department (2019). Health Disparity and Health Inequity: 2019 Trends and Data Report Houston/Harris County Summary Report.

[10] Ordonez E, Dowdell K, Navejar NM (2021). An assessment of the social determinants of health in an urban emergency department. West J Emerg Med.

[11] Harris County Public Health (2021). COVID-19 Vaccine Distribution.

[12] Kwok KO, Lai F, Wei WI (2020). Herd immunity – estimating the level required to halt the COVID-19 epidemics in affected countries. J Infect.

[13] Hotez PJ (2020). COVID-19 meets the antivaccine movement. Microbes Infect.

[14] Kaiser Family Foundation (2021). Early state vaccination data raise warning flags for racial equity.

[15] Kinder Institute for Urban Research (2021). Mapping inequity in Houston’s COVID-19 vaccination rollout.

[16] Romer D, Jamieson KH (2020). Conspiracy theories as barriers to controlling the spread of COVID-19 in the U.S. Soc Sci Med.

[17] Morley C, Unwin M, Peterson GM (2018). Emergency department crowding: a systematic review of causes, consequences and solutions. PLoS One.

[18] Lin C, Tu P, Beitsch LM (2020). Confidence and receptivity for COVID-19 vaccines: a rapid systematic review. Vaccines (Basel).

[19] Gamble VN (1997). Under the shadow of Tuskegee: African Americans and health care. Am J Public Health.

[20] Skloot R (2011). The Immortal Life of Henrietta Lacks.

[21] Roberts D (1998). Killing the Black Body: Race, Reproduction, and the Meaning of Liberty.

[22] Khubchandani J, Macias Y (2021). COVID-19 vaccination hesitancy in Hispanics and African-Americans: a review and recommendations for practice. Brain Behav Immun Health.

[23] Shen MJ, Peterson EB, Costas-Muñiz R (2018). The effects of race and racial concordance on patient-physician communication: a systematic review of the literature. J Racial Ethn Health Disparities.

